# Maternal Consumption of Fruits, Vegetables and Fruit Juices in Relation to Offspring Weight at 4 Years

**DOI:** 10.3390/nu18172918

**Published:** 2026-09-06

**Authors:** Lucía Riggioni-Saborío, Cristina Jardí, Nerea Becerra-Tomás, Josué Cruz-Rodríguez, Victoria Arija

**Affiliations:** 1 Nutrition and Mental Health (NUTRISAM) Research Group, Unitat de Salut Pública i Epidemiologia Nutricional, Departament de Ciències Mèdiques Bàsiques, Universitat Rovira i Virgili, 43201 Reus, Spain; lucia.riggioni@urv.cat (L.R.-S.); cristina.jardi@urv.cat (C.J.); nerea.becerra@urv.cat (N.B.-T.); josue.cruz@urv.cat (J.C.-R.); 2 Institut de Recerca Biomèdica Catalunya Sud (IRB CatSud), 43005 Tarragona, Spain; 3 Collaborative Group on Lifestyles, Nutrition, and Tobacco (CENIT), Institut D’Investigació en Atenció Primària IDIAP Jordi Gol, 08007 Barcelona, Spain

**Keywords:** maternal nutrition, pregnancy, fruit consumption, vegetable consumption, fruit juice consumption, childhood obesity

## Abstract

**Background/Objectives**: Maternal diet plays a crucial role in fetal development and may influence offspring weight development. Although fruits and vegetables provide nutritional and metabolic benefits during pregnancy, evidence regarding their specific role on childhood overweight/obesity remains limited. This study aimed to examine the association between maternal intake of fruits, vegetables, and fruit juices during pregnancy and the risk of overweight/obesity in children at 4 years of age. **Methods**: We analyzed 252 mother–child pairs from the Mediterranean ECLIPSES cohort in a prospective observational study. Maternal dietary intake was assessed at 12, 24 and 36 weeks of gestation using a semiquantitative food frequency questionnaire previously validated in a Spanish population. Childhood overweight/obesity was defined as a BMI z-score above the 85th percentile for age and sex at age 4. Multivariable-adjusted logistic regression models estimated ORs and 95% CIs. **Results**: Higher maternal consumption of fruits and vegetables was inversely associated with overweight/obesity in children (OR: 0.31 [0.13–0.74]). No associations were observed for total or natural fruit juice, whereas higher consumption of commercial fruit juice was associated with increased odds of overweight/obesity (OR: 2.48 [1.06–5.78]). **Conclusions**: Higher maternal combined consumption of fruits and vegetables during pregnancy was associated with lower odds of overweight or obesity in offspring at 4 years of age, whereas the opposite association was observed for commercial, but not natural, fruit juice. These associations may involve both prenatal biological and postnatal behavioral and family dietary pathways; however, the commercial fruit juice finding should be considered exploratory and requires confirmation in larger prospective studies.

## 1. Introduction

Childhood obesity has become a major global public health challenge, with a steadily increasing prevalence over recent decades [1,2]. In Europe, Spain has the second-highest prevalence [3]. This condition is associated with adverse psychosocial outcomes and a reduced quality of life [4,5], as well as an increased risk of chronic diseases later in life, including cardiovascular disease [5,6], type 2 diabetes [4,5], metabolic syndrome, and certain cancers [5,7].

Given the growing burden of childhood obesity and its long-term health consequences, effective prenatal preventive strategies targeting modifiable maternal factors are essential. The first 1000 days of life, starting from conception, represent a critical window for growth and development, during which modifiable factors can shape future health outcomes [8]. Among these, maternal lifestyle factors during pregnancy, including diet, physical activity, smoking, and alcohol consumption, have been identified as important determinants of offspring health [9]. Maternal diet is especially relevant, as it shapes the intrauterine environment and influences fetal programming, potentially affecting the long-term risk of obesity in the offspring [10,11].

Previous research on maternal diet and childhood overweight/obesity has primarily focused on adherence to healthy dietary patterns. Greater adherence during pregnancy to healthy dietary patterns characterized by a high intake of fruits and vegetables, such as the Mediterranean diet (MedDiet) and the Dietary Approaches to Stop Hypertension (DASH) diet, has been associated with more favorable offspring growth trajectories, lower adiposity, and a reduced risk of childhood overweight/obesity [10,12,13,14,15]. One of the defining characteristics of these healthy dietary patterns is a high consumption of fruits and vegetables, which are important sources of essential vitamins, minerals, dietary fiber, and bioactive compounds with antioxidant and anti-inflammatory properties [16,17]. These nutritional and bioactive components may influence maternal metabolic health and the intrauterine environment [18,19], thereby affecting fetal growth and development [20,21], with potential long-term implications for offspring adiposity [10,11].

Beyond prenatal biological mechanisms, maternal diet may also influence childhood body weight through behavioral and family-related pathways. Maternal dietary habits and preferences may persist after pregnancy and shape children’s food preferences and dietary intake through early flavor exposure, parental modeling, and the home food environment [22]. Previous evidence suggests that higher maternal diet quality during pregnancy is associated with greater fruit and vegetable intake in early childhood, an association that may partly reflect the continuity of maternal dietary habits after birth [23]. Thus, both prenatal and postnatal pathways may contribute to the association between maternal diet during pregnancy and subsequent childhood body weight. Although healthy dietary patterns have been associated with childhood overweight/obesity, studies specifically investigating fruit and vegetable consumption have mainly focused on fetal and early growth outcomes, reporting associations with a lower risk of being small for gestational age (SGA) [16,24,25] and improved fetal and infant growth up to six months of age [26,27]. In addition, only two prospective studies have evaluated maternal fruit juice consumption during pregnancy in relation to offspring adiposity or overweight/obesity, with inconsistent findings. These studies assessed fruit juice as a single exposure, without distinguishing between natural and commercial fruit juice [28,29].

To our knowledge, no prospective study has comprehensively evaluated the association between maternal consumption of fruits, vegetables, and different types of fruit juice during pregnancy and the risk of childhood overweight/obesity in offspring.

To address this knowledge gap, we analyzed data from the Mediterranean ECLIPSES cohort of healthy pregnant women and their children. The aim of this study was to examine the associations between maternal consumption of fruits, vegetables, and both natural and commercial fruit juices during pregnancy and the risk of overweight/obesity in offspring at 4 years of age.

## 2. Materials and Methods

### 2.1. Study Design and Participants

The present study was conducted within the framework of the ECLIPSES trial, a community-based randomized controlled trial carried out in Tarragona, Catalonia, Spain. Eligible participants were healthy women aged ≥18 years with a gestational age (GA) of ≤12 weeks. Full details of the study, including the inclusion and exclusion criteria, have been described elsewhere [30]. The ECLIPSES study is registered at www.clinicaltrialsregister.eu (number EUCTR-2012–005480-28; accessed on 5 September 2026) and at www.clinicaltrials.gov (number NCT03196882; accessed on 5 September 2026).

**Figure 1:**
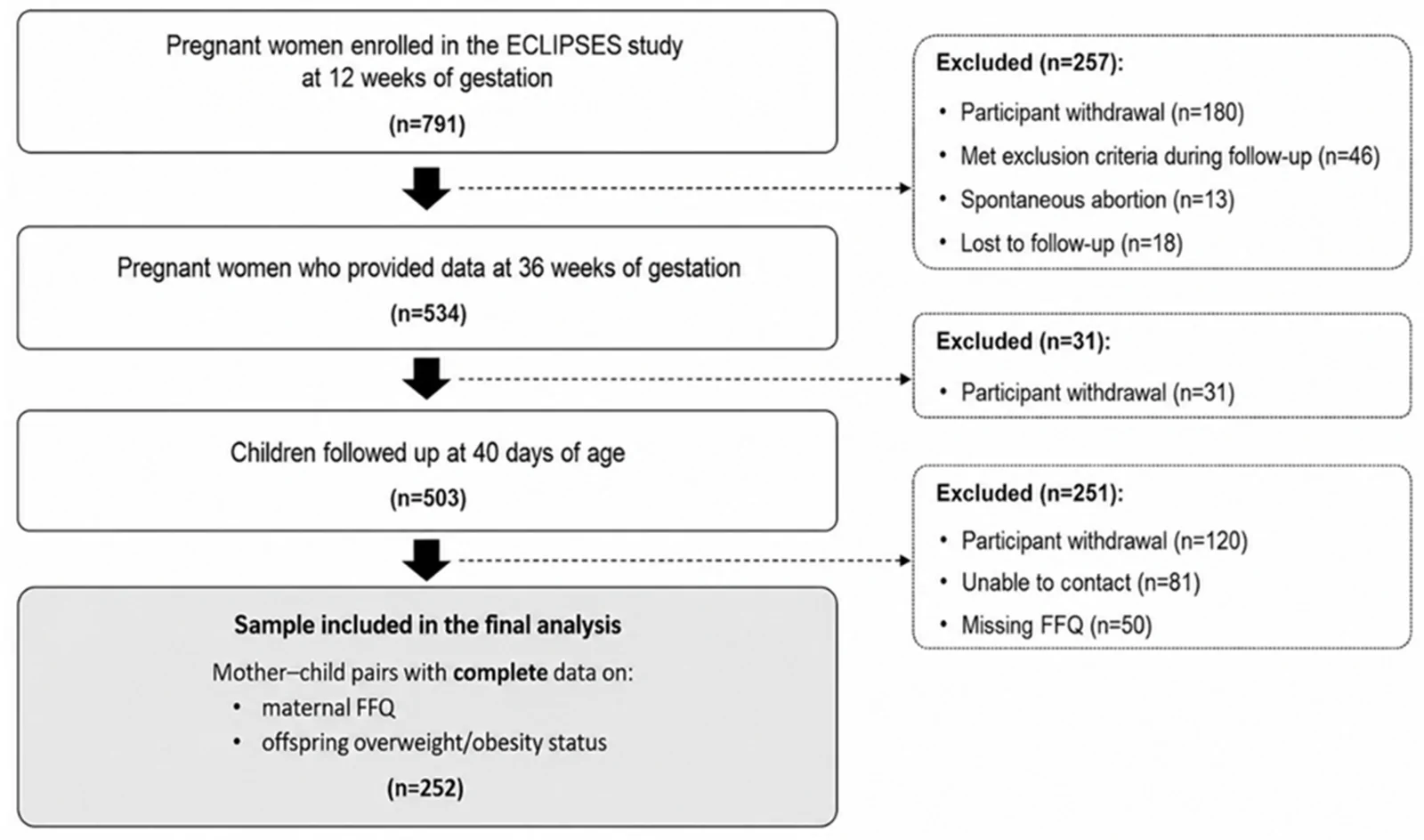
Flowchart of the participants in the study. ECLIPSES, Ensayo CLInico Para Suplementar con hierro a EmbarazadaS; FFQ, food frequency questionnaire.

The current analysis is based on a longitudinal study design, using data from a subsample of healthy pregnant women from the ECLIPSES trial and data collected from their children at 4 years of age. Only participants with available data on food frequency questionnaire (FFQ) and child overweight/obesity status at 4 years were included in the present prospective analyses, resulting in a final analytical sample of 252 mother–child pairs (Figure 1).

### 2.2. Dietary Assessment

Maternal dietary habits were assessed using a semi-quantitative 45-item FFQ previously validated in a Spanish population [31], administered at 12, 24, and 36 weeks of gestation. The FFQ included multiple food groups and was not restricted to the dietary exposures examined in the present study.

Participants reported their usual frequency of consumption for each FFQ item, either weekly or monthly. Reported consumption frequencies were converted to daily frequencies by dividing weekly frequencies by 7 and monthly frequencies by 30. Daily intake (g/day) of each item was then calculated by multiplying the daily consumption frequency by the corresponding standard portion size. Standard portion sizes were derived from population-based consumption data from the Food Consumption Estimation study (ECA-REF) [32,33]. For the dietary exposures examined in the present study, the portion sizes used were 100 g for fruit items, 30 g for salads, 100 g for green beans, Swiss chard and spinach, 50 g for side vegetables, and 200 g for both natural and commercial fruit juice. Total daily energy intake was estimated from the complete FFQ using the REGAL (Répertoire Général des Aliments) [34] and Mataix Verdú Spanish [35] food composition tables.

The FFQ included separate items for fruits, vegetables, and fruit juices. Fruit consumption was assessed using FFQ items for citrus fruits (e.g., orange, tangerine) and other fruits (e.g., apple, pear, peach, apricot, banana).

Vegetable consumption was assessed using three FFQ items: salads (e.g., lettuce, tomato, endive); green beans, Swiss chard, spinach; and side vegetables (e.g., eggplant, zucchini, mushrooms). The individual fruits and vegetables listed above are examples of foods included within the corresponding FFQ items and do not represent an exhaustive list of the fruits and vegetables assessed.

Fruit juice consumption was assessed using separate FFQ items for natural fruit juice and commercial fruit juice. Natural fruit juice referred to homemade juice prepared from fresh fruit, whereas commercial fruit juice included all commercially produced or packaged fruit juice products, irrespective of whether they contained added sugars, including 100% packaged fruit juice, reconstituted juice from concentrate, nectars, and fruit drinks. These commercial fruit juice subtypes were not assessed separately, and no additional instructions were provided to participants regarding their classification. Participants reported their habitual consumption according to the broad distinction between homemade and commercial fruit juice established in the validated FFQ.

Vegetable juice consumption was not assessed as a separate item in the FFQ and therefore could not be evaluated as an independent dietary exposure.

For all dietary variables, the average consumption across the three trimesters of pregnancy was calculated and adjusted for total energy intake using the residual method [36].

### 2.3. Outcome

At age 4, children were assessed in person by trained nutritionists. Weight was measured using a TANITA digital scale (Body Composition Analyzer TANITA BC-418, Tanita Corporation of America, Inc., Arlington Heights, IL, USA) with children wearing light clothing and no shoes. Height was measured to the nearest millimeter using a portable stadiometer (SECA 222^®^, Hamburg, Germany), following the Frankfurt horizontal plane standard. All measurements were performed in duplicate, and the average was used for analysis.

BMI (kg/m^2^) was calculated from measured weight and height and converted to BMI-for-age z-scores according to the WHO Child Growth Standards for children aged 0–5 years [37]. The dependent variable of this study was childhood overweight and/or obesity at age 4, defined as a BMI z-score above the 85th percentile for age and sex [38].

### 2.4. Other Covariates Assessment

Baseline maternal characteristics included sociodemographic variables (maternal age and educational level) and lifestyle-related variables (smoking status). Maternal educational level was categorized into primary/secondary and university studies.

Maternal height and weight were measured at enrollment and throughout pregnancy. Based on their initial BMI, women were classified as having normal weight, overweight, or obesity [39].

Adherence to the Mediterranean Diet (MedDiet) was assessed using the relative MedDiet (rMedDiet) score, adapted from the original MedDiet score developed by Trichopoulou et al. [40,41,42]. For each dietary component (except alcohol), intake was expressed as energy density (g/1000 kcal/day) and categorized into tertiles based on the study population distribution. For beneficial components (nuts, legumes, cereals, fresh fish, and olive oil), scores of 0, 1, and 2 points were assigned to the lowest, middle, and highest tertiles, respectively, whereas scoring was reversed for non-adherent components (meat and dairy products, with 2 points assigned to the lowest tertile and 0 to the highest). Alcohol consumption was scored as 2 points for total abstinence and 0 points for any intake. The original rMedDiet score includes nine dietary components (range: 0–18 points), including fruits and vegetables [40,41,42]. However, in the present study, fruits and vegetables were excluded from the score to avoid collinearity and overadjustment, as they constituted the primary exposures of interest. The modified rMedDiet score therefore comprised seven dietary components, with scores ranging from 0 to 14 points. Child-related variables included GA at birth (weeks), birth weight (g), and sex.

### 2.5. Statistical Analysis

Participants were categorized into exposure-specific tertiles according to their fruit, vegetable and fruit juice consumption. Descriptive data are presented as means (±SD) for continuous variables and as numbers (%) for categorical variables. Chi-square tests (for categorical variables) and one-way ANOVA (for continuous variables) were used to compare maternal and child characteristics across tertiles of fruit, vegetable and fruit juice consumption. Baseline maternal characteristics were also compared between participants included in the analytical sample and those not included using standardized mean differences (SMDs), in addition to significance tests, to assess potential differences related to attrition (Supplementary Table S1).

Multivariable logistic regression models were used to estimate odds ratios (OR) and 95% confidence intervals (CIs) for childhood overweight/obesity.

Covariate selection for the multivariable logistic regression models was guided by a directed acyclic graph (DAG) based on an a priori causal framework (Supplementary Figure S1). The final adjustment set included maternal age (<25, 25 to <30, ≥30 years), maternal initial BMI (kg/m^2^), maternal educational level (primary/secondary, university), smoking status (yes/no), total energy intake (kcal/d) and adherence to the MedDiet (rMedDiet score). Among the covariates included in the multivariable models, missing data were present only for the rMedDiet score (n = 12; 4.8%) and were imputed using the mean value. As a sensitivity analysis, multivariable models were repeated using complete cases for the rMedDiet score.

To assess the potential impact of differential attrition, an inverse-probability-of-attrition weighting (IPAW) sensitivity analysis was performed. The probability of inclusion in the analytical sample was estimated among the initially enrolled participants using a logistic regression model including baseline maternal age, initial BMI, educational level, smoking status, and total energy intake. Stabilized inverse-probability weights were calculated and applied to the multivariable logistic regression models. The IPAW-weighted models included the same covariate adjustment set as the primary models and used robust variance estimation. The stabilized weights had a mean of 0.996 and ranged from 0.44 to 2.25.

The study evaluated maternal fruit, vegetable, and fruit juice consumption as related dietary exposures, with no single dietary exposure designated a priori as the primary exposure. Given the evaluation of multiple correlated dietary exposures and comparisons, findings for specific juice types were considered exploratory. To assess linear trends, the median value of the exposure variable within each tertile was assigned to all participants in that tertile and included in the models as a continuous variable.

In additional analyses, dietary exposures were modeled as continuous variables to estimate ORs and 95% CIs for childhood overweight/obesity per 100-g/day increment in maternal intake. Potential departures from linearity were assessed by additionally including a quadratic term for each dietary exposure in the corresponding multivariable model.

All analyses were performed using SPSS software, version 29.0.

## 3. Results

### 3.1. Characteristics of the Study Population

Among the 252 mother-–child pairs included in the analysis, 24.6% of children had overweight or obesity. Baseline maternal characteristics of participants included in the analytical sample (n = 252) and those not included (n = 539) are presented in Supplementary Table S1. The largest differences were observed for maternal educational level (SMD = 0.51) and age (SMD = 0.37), with included participants having a higher educational level and being older than those not included. Differences in maternal initial BMI (SMD = 0.01), smoking status (SMD = 0.00), and total energy intake (SMD = 0.09) were small or negligible.

Table 1 presents the baseline characteristics of the study population according to tertiles of fruit, vegetable and fruit juice consumption. Across tertiles of fruit consumption, women in the highest tertile were less likely to smoke than those in the lowest tertile. Across tertiles of vegetable consumption, women in the highest tertile were older than those in the lowest tertile. No significant differences in maternal or child characteristics were observed across tertiles of total fruit juice consumption.

Mean daily intakes of fruits, vegetables, and fruit juice were 119.03, 74.47, and 104.48 g/day, respectively. Natural fruit juice represented 55% of total fruit juice intake.

### 3.2. Associations Between Maternal Fruit, Vegetable, and Fruit Juice Consumption and Childhood Overweight/Obesity

As shown in Table 2, higher maternal combined fruit and vegetable consumption was inversely associated with offspring overweight/obesity (OR T3 vs. T1: 0.31; 95% CI: 0.13–0.74; *p*-trend = 0.01). When analyzed separately, fruit and vegetable consumption were not significantly associated with overweight/obesity (OR T3 vs. T1: 0.47; 95% CI: 0.21–1.03; *p*-trend = 0.06, and OR T3 vs. T1: 0.48; 95% CI: 0.21–1.11; *p*-trend = 0.08, respectively). Total and natural fruit juice consumption were not associated with overweight/obesity at 4 years of age. In contrast, higher commercial fruit juice consumption was associated with increased odds of overweight/obesity (OR T3 vs. T1: 2.48; 95% CI: 1.06–5.78; *p*-trend= 0.03). Given the number of correlated dietary exposures and comparisons evaluated, this finding should be interpreted cautiously and considered exploratory and hypothesis-generating.

In the IPAW sensitivity analysis, estimates were broadly consistent with those from the primary multivariable analyses (Supplementary Table S2). The inverse association for the highest versus lowest tertile of combined fruit and vegetable consumption remained statistically significant (OR: 0.36; 95% CI: 0.14–0.93), as did the positive association for commercial fruit juice consumption (OR: 3.37; 95% CI: 1.27–8.93). Similarly, complete-case analyses excluding participants with missing rMedDiet scores yielded materially similar estimates to those obtained in the primary analyses.

In additional analyses modeling dietary exposures continuously, none of the adjusted associations per 100-g/day increment reached statistical significance (Table S3). The direction of the estimates was generally consistent with the tertile-based analyses, with inverse associations for combined fruit and vegetable consumption, fruit consumption, and vegetable consumption, and a positive association for commercial fruit juice consumption. No evidence of departure from linearity was observed, as none of the quadratic terms were statistically significant (all *p* > 0.05).

## 4. Discussion

### 4.1. Principal Findings and Comparison with Previous Studies

The present prospective study found that higher maternal consumption of fruits and vegetables during pregnancy was associated with lower odds of childhood overweight/obesity in offspring. In contrast, neither total nor natural fruit juice consumption was associated with offspring overweight/obesity. However, higher intake of commercial fruit juice was associated with increased odds of overweight/obesity in offspring.

Our findings contribute to the growing body of evidence highlighting the importance of maternal diet during pregnancy for offspring health outcomes [10,11,43]. Previous studies investigating maternal fruit and vegetable consumption have reported associations with improved anthropometric outcomes in offspring, particularly a lower risk of being SGA [16,24,25] and improved fetal and infant growth up to 6 months of age [26,27]. In contrast, research examining childhood overweight and obesity has largely focused on overall dietary patterns rather than specific food groups. Higher adherence to healthy dietary patterns characterized by a high intake of fruits and vegetables, such as the MedDiet and the DASH diet, has been associated with more favorable offspring growth trajectories, lower adiposity, and a reduced risk of childhood overweight/obesity [10,12,13,14,15]. However, the specific contribution of fruits and vegetables to the beneficial associations observed for these dietary patterns remains unclear. To our knowledge, this is among the first prospective studies to specifically evaluate the association between maternal fruit, vegetable and fruit juice consumption during pregnancy and offspring overweight/obesity risk.

### 4.2. Potential Prenatal and Postnatal Pathways

Several biological mechanisms may support the plausibility of our findings. Fruits and vegetables are rich in vitamins, minerals, dietary fiber, and bioactive compounds with antioxidant and anti-inflammatory properties, including polyphenols, carotenoids, and phytosterols [18]. These nutrients and bioactive compounds may improve maternal metabolic health by reducing oxidative stress, systemic inflammation, and metabolic dysregulation during pregnancy, which may contribute to a more favorable intrauterine environment for fetal development [18,19]. In addition, their high fiber content and generally low glycemic load may contribute to improved glycemic control during pregnancy [44,45]. Maternal metabolic status during pregnancy is increasingly recognized as an important determinant of fetal metabolic programming, with potential long-term implications for obesity and other metabolic disorders in offspring [46]. Collectively, these pathways provide biological plausibility for the inverse association observed between maternal combined fruit and vegetable consumption and childhood overweight/obesity, although the present observational study cannot establish the mechanism underlying this association.

However, prenatal biological programming may not be the only pathway potentially linking maternal fruit and vegetable consumption during pregnancy with childhood body weight. Maternal diet may contribute to the development of offspring food preferences through early flavor exposure, while maternal dietary habits and preferences may persist after pregnancy and shape children’s dietary intake through parental modeling and the home food environment [22]. Previous evidence suggests that higher maternal diet quality during pregnancy is associated with greater fruit and vegetable intake in early childhood and that this association may partly reflect the stability of maternal dietary habits from pregnancy into the postnatal period [23]. Therefore, the inverse associations observed in the present study may reflect not only prenatal biological pathways but also the continuity of maternal and family dietary behaviors after birth. However, as longitudinal information on children’s dietary intake and the postnatal food environment was not available in the present analysis, these pathways could not be directly evaluated.

Previous evidence on maternal fruit juice consumption during pregnancy and childhood overweight/obesity is limited to two prospective studies, which reported inconsistent results [28,29]. Notably, neither study evaluated natural and commercial fruit juice as separate dietary exposures. Our findings for total fruit juice are consistent with those from the Project Viva study [28], but differ from the Generation R Study, which reported a positive association with offspring overweight/obesity [29]. Importantly, because previous studies evaluated fruit juice as a single dietary exposure, direct comparisons with our findings for natural and commercial fruit juice separately are not possible.

When fruit juice was analyzed according to its type, no association was observed for natural fruit juice, whereas higher commercial fruit juice consumption was associated with increased odds of childhood overweight/obesity. Given the multiple correlated dietary exposures and comparisons evaluated, this finding should be interpreted cautiously and considered exploratory and hypothesis-generating.

Although natural fruit juices retain naturally occurring sugars, vitamins, minerals, and bioactive compounds such as polyphenols, they contain substantially less dietary fiber than whole fruits [47]. This nutritional difference may partly explain why no protective association was observed for natural fruit juice in our study.

Several pathways may potentially contribute to the exploratory association observed for commercial fruit juice. Compared with natural fruit juice, commercially available fruit juices may differ in their nutritional composition because of industrial processing and formulation. A previous compositional analysis reported higher sucrose and total carbohydrate contents, together with differences in micronutrient composition, in commercially available fruit juices compared with natural fruit juices [48]. Although the biological implications of these compositional differences remain uncertain, they may partly contribute to the association observed in our study. However the heterogeneity of the commercial fruit juice should be considered when interpreting this association. In addition, prenatal exposure to flavors from the maternal diet may influence offspring taste preferences and promote greater acceptance of sweet-tasting foods after birth [29]. Beyond these potential prenatal mechanisms, maternal beverage consumption during pregnancy may also reflect broader family dietary and lifestyle patterns that persist after birth, which could further contribute to childhood obesity risk [28]. However, these pathways were not directly assessed in the present study, and the mechanisms underlying the observed association remain uncertain.

### 4.3. Strengths and Limitations

While our study provides valuable insights, it is not without limitations. First, as an observational study, causal relationships cannot be established, and residual confounding by unmeasured maternal, family, and postnatal factors cannot be excluded. Relevant factors not accounted for in the present analyses include gestational diabetes, paternal BMI, and postnatal characteristics such as the child’s diet and beverage consumption, physical activity, and socioeconomic environment. The lack of longitudinal information on children’s dietary intake during early childhood also limited our ability to evaluate the potential contribution of postnatal dietary pathways. Second, substantial loss to follow-up occurred between initial recruitment and the 4-year assessment. Participants included in the analytical sample differed from those not included, particularly in maternal age and educational level, raising the possibility of selection bias due to differential retention. In addition, the primary analyses were restricted to participants with available exposure and outcome data, which may have introduced additional selection bias if data availability was related to characteristics associated with the exposure or outcome. However, the IPAW sensitivity analysis yielded estimates broadly consistent with those from the primary analyses, supporting the robustness of the main findings to differential attrition related to the measured baseline characteristics. Nevertheless, residual selection bias cannot be completely ruled out.

Third, despite the use of a parsimonious adjustment set based on an a priori causal framework, the relatively limited number of outcome events may have increased the risk of overfitting. The final models included 62 overweight/obesity events and nine exposure and covariate parameters, corresponding to approximately 6.9 events per parameter. However, the main estimates remained similar after adopting the more parsimonious model, providing some reassurance regarding their stability to model specification. Furthermore, although the continuous analyses per 100-g/day increment did not reach statistical significance, the direction of the estimates was generally consistent with the tertile-based analyses. Differences in statistical significance may partly reflect differences in exposure parameterization. No evidence of departure from linearity was observed, as none of the quadratic terms were statistically significant. In addition, given the number of correlated dietary exposures and comparisons evaluated, the possibility of chance findings due to multiple testing cannot be excluded. Therefore, particularly the findings for commercial fruit juice should be interpreted cautiously and considered exploratory and hypothesis-generating.

Fourth, maternal dietary intake was assessed using a semiquantitative FFQ, which is subject to recall bias and measurement error. However, the FFQ was previously validated in a Spanish population, supporting the reliability of the dietary assessment.

Finally, the findings may not be generalizable to other populations, as the study included healthy pregnant women from the Mediterranean region with specific sociodemographic characteristics and dietary habits.

Despite these limitations, the study also presents several strengths. The prospective design and adjustment for relevant potential confounders based on an a priori causal framework strengthen the methodological rigor of the study. In addition, we evaluated fruits, vegetables, their combined consumption, and different types of fruit juice separately, allowing a more comprehensive assessment of their individual associations with childhood overweight/obesity. Furthermore, dietary intake was assessed at three time points during pregnancy (first, second, and third trimesters), which allowed us to estimate average dietary intake across pregnancy and thereby reduce the influence of within-person variation.

## 5. Conclusions

Higher maternal combined consumption of fruits and vegetables was associated with lower odds of childhood overweight/obesity at age 4. In contrast, no significant associations were observed for total or natural fruit juice, whereas higher commercial fruit juice consumption was associated with increased odds of childhood overweight/obesity. Given the multiple dietary comparisons performed, the finding for commercial fruit juice should be considered exploratory and hypothesis-generating. The observed associations may involve both prenatal biological pathways related to the maternal metabolic and intrauterine environment and postnatal behavioral and family dietary pathways; however, these mechanisms could not be directly evaluated in the present study. Further prospective studies in larger cohorts, with appropriate consideration of maternal, family, and postnatal factors, are needed to confirm these findings and better understand the underlying pathways. These findings should therefore not yet be considered a basis for specific dietary recommendations during pregnancy.

## Figures and Tables

**Table 1:** Baseline characteristics of the study population according to tertiles of fruit, vegetable and fruit juice consumption.

	Fruit Consumption, g/d				Vegetable Consumption, g/d				Fruit juice Consumption, g/d			
	T1 (≤97)	T2 (98–135)	T3 (≥136)	*p*-Value	T1 (≤58)	T2 (59–80)	T3 (≥81)	*p*-Value	T1 (≤71)	T2 (72–126)	T3 (≥127)	*p*-Value
Maternal characteristics												
Age, years, mean ± SD	31.08 ± 4.36	31.81 ± 4.36	31.86 ± 4.47	0.468	30.48 ± 4.53	31.35 ± 4.34	32.93 ± 4.48	**0.002**	31.94 ± 4.56	31.08 ± 4.27	31.73 ± 4.82	0.448
Initial BMI, kg/m^2^, mean ± SD	25.50 ± 4.50	24.70 ± 4.44	24.87 ± 4.66	0.482	25.57 ± 4.43	24.66 ± 4.69	24.84 ± 4.47	0.395	25.37 ± 4.59	25.08 ± 5.02	24.62 ± 3.94	0.557
Education, n (%)												
Primary/Secondary	51 (60.7)	42 (50.0)	47 (56.0)	0.375	55 (65.5)	44 (52.4)	41 (48.8)	0.073	49 (58.3)	48 (57.1)	43 (51.2)	0.608
University	33 (39.3)	42 (50.0)	37 (44.0)		29 (34.5)	40 (47.6)	43 (51.2)		35 (41.7)	36 (42.9)	41 (48.8)	
Smoking status, n (%)												
Smoker	23 (27.4)	10 (11.9)	12 (14.3)	**0.019**	20 (23.8)	12 (14.3)	13 (15.5)	0.214	20 (23.8)	15 (17.9)	10 (11.9)	0.131
Mode of delivery, n (%)												
Vaginal	68 (81.0)	67 (79.8)	65 (77.4)	0.844	67 (79.8)	70 (83.3)	63 (75.0)	0.408	66 (78.6)	66 (78.6)	68 (81.0)	0.908
Cesarean section	16 (19.0)	17 (20.2)	19 (22.6)		17 (20.2)	14 (16.7)	21 (25.0)		18 (21.4)	18 (21.4)	16 (19.0)	
TEI, kcal/d, mean ± SD	2139.81 ± 496.10	2006.37 ± 397.64	2073.59 ± 436.24	0.154	2090.68 ± 429.26	1994.67 ± 428.80	2134.58 ± 474.42	0.116	2106.68 ± 416.16	1987.85 ± 458.16	2125.25 ± 457.40	0.096
MedDiet score during pregnancy, points (14-points), mean ± SD	8 ± 2	8 ± 2	8 ± 2	0.964	8 ± 2	8 ± 2	8 ± 2	0.109	8 ± 2	8 ± 2	8 ± 2	0.223
Stratum 1 (Hb 110–130 g/L), n (%)												
Iron supplementation, 40 mg/day	27 (54.0)	26 (44.8)	29 (50.9)	0.621	24 (40.7)	27 (48.2)	31 (62.0)	0.082	19 (40.4)	32 (57.1)	31 (50.0)	0.239
Iron supplementation, 80 mg/day	23 (46.0)	32 (55.2)	28 (49.1)		35 (59.3)	29 (51.8)	19 (38.0)		28 (59.6)	24 (42.9)	31 (50.0)	
Stratum 2 (Hb > 130 g/L), n (%)												
Iron supplementation, 20 mg/day	16 (47.1)	11 (42.3)	15 (55.6)	0.618	12 (48.0)	16 (57.1)	14 (41.2)	0.456	16 (43.2)	16 (57.1)	10 (45.5)	0.515
Iron supplementation, 40 mg/day	18 (52.9)	15 (57.7)	12 (44.4)		13 (52.0)	12 (42.9)	20 (58.8)		21 (56.8)	12 (42.9)	12 (54.5)	
Child characteristics												
Sex, male, n (%)	41 (48.8)	48 (57.1)	38 (45.2)	0.285	41 (48.8)	45 (53.6)	41 (48.8)	0.776	40 (47.6)	42 (50.0)	45 (53.6)	0.740
Birth weight, grams, mean ± SD	3301.19 ± 472.10	3263.09 ± 424.55	3252.42 ± 434.33	0.756	3289.16 ± 451.06	3248.38 ± 477.81	3379.15 ± 400.53	0.825	3255.75 ± 384.33	3224.78 ± 427.33	3336.16 ± 506.05	0.244
GA at delivery, weeks, mean ± SD	39.85 ± 1.18	39.86 ± 1.43	39.38 ± 1.88	0.071	39.95 ± 1.26	39.49 ± 1.98	39.64 ± 1.22	0.135	39.67 ± 1.36	39.71 ± 1.60	39.70 ± 1.65	0.978
BMI-for-age z-score at 4 years, mean ± SD	0.60 ± 1.36	0.41 ± 1.28	0.20 ± 1.23	0.135	0.52 ± 1.21	0.53 ± 1.44	0.17 ± 1.20	0.122	0.36 ± 1.42	0.60 ± 1.47	0.26 ± 0.93	0.213

Values are means ± SD or number (%) for continuous and categorical variables, respectively. T, tertile; SD, standard deviation; BMI, body mass index; IOM, Institute of Medicine; TEI, total energy intake; MedDiet, Mediterranean diet; Hb, hemoglobin; GA, gestational age. Bold *p*-values indicate statistical significance (*p* < 0.05).

**Table 2:** Multivariate-adjusted odds ratios and 95% confidence intervals for childhood overweight/obesity at 4 years of age according to tertiles fruit, vegetable, and fruit juice consumption.

Tertiles of Consumption in g/d				
	T1 (Lowest)	T2 (n = 84)	T3 (Highest)	*p*-Trend
Fruit and vegetable consumption, median (Q1–Q3)	125.93 (102.78–143.90)	187.29 (172.95–193.50)	260.59 (231.34–302.40)	
Overweight/obesity prevalence, n (%)	28 (33.3)	21 (25.0)	13 (15.5)	
Crude model	1 (Ref.)	0.67 (0.34, 1.30)	0.37 (0.17, 0.77)	0.08
Multivariable model	1 (Ref.)	0.73 (0.33, 1.63)	0.31 (0.13, 0.74)	**0.01**
Fruit consumption, median (Q1–Q3)	64.67 (45.23–79.78)	119.03 (106.23–123.84)	170.22 (149.18–195.54)	
Overweight/obesity prevalence, n (%)	28 (33.3)	18 (21.4)	16 (19.0)	
Crude model	1 (Ref.)	0.54 (0.27, 1.09)	0.47 (0.23, 0.96)	**0.03**
Multivariable model	1 (Ref.)	0.60 (0.27, 1.33)	0.47 (0.21, 1.03)	0.06
Vegetable consumption, median (Q1–Q3)	47.73 (31.35–52.41)	70.53 (63.64–74.47)	100.90 (88.76–125.26)	
Overweight/obesity prevalence, n (%)	26 (31.0)	22 (26.2)	14 (16.7)	
Crude model	1 (Ref.)	0.79 (0.40, 1.55)	0.45 (0.21, 0.93)	**0.03**
Multivariable model	1 (Ref.)	0.96 (0.45, 2.06)	0.48 (0.21, 1.11)	0.08
Total fruit juice consumption, median (Q1–Q3)	44.92 (22.72–62.72)	100.29 (84.76–104.50)	158.60 (139.25–205.39)	
Overweight/obesity prevalence, n (%)	19 (22.6)	28 (33.3)	15 (17.9)	
Crude model	1 (Ref.)	1.71 (0.86, 3.39)	0.74 (0.35, 1.58)	0.45
Multivariable model	1 (Ref.)	2.10 (0.94, 4.66)	0.99 (0.43, 2.30)	0.98
Natural fruit juice consumption, median (Q1–Q3)	10.29 (1.54–19.07)	52.23 (38.31–57.39)	98.11 (80.66–140.06)	
Overweight/obesity prevalence, n (%)	26 (31.0)	18 (21.4)	18 (21.4)	
Crude model	1 (Ref.)	0.61 (0.30, 1.22)	0.61 (0.30, 1.22)	0.16
Multivariable model	1 (Ref.)	0.74 (0.33, 1.65)	0.65 (0.29, 1.45)	0.29
Commercial fruit juice consumption, median (Q1–Q3)	6.86 (0.69–13.12)	38.38 (29.72–46.16)	90.93 (66.44–122.58)	
Overweight/obesity prevalence, n (%)	16 (19.0)	22 (26.2)	24 (28.6)	
Crude model	1 (Ref.)	1.51 (0.73, 3.13)	1.70 (0.83, 3.50)	0.18
Multivariable model	1 (Ref.)	1.34 (0.55, 3.27)	2.48 (1.06, 5.78)	**0.03**

OR, odds ratios; T, tertile; CI, confidence interval; Ref., reference category. Multivariable model adjusted for mother’s age (<25, 25 to <30 or ≥30 years), mother’s initial BMI (kg/m^2^), mother’s education level (primary/secondary or university), smoking status (yes/no), total energy intake (kcal/d), and modified 14-point rMedDiet score excluding fruits and vegetables (continuous variable). Bold *p*-values indicate statistical significance (*p* < 0.05).

## Data Availability

The data presented in this study are available on request from the corresponding author.

## Supplementary Materials

The following supporting information can be downloaded at: https://www.mdpi.com/article/10.3390/nu18172918/s1, Figure S1: Directed acyclic graph (DAG) for the association between maternal intake of fruits, vegetables, and fruit juices during pregnancy and childhood overweight/obesity at 4 years; Table S1: Baseline maternal characteristics of participants included and not included in the analytical sample; Table S2: Sensitivity analysis using inverse-probability-of-attrition weighting (IPAW) for the associations between maternal fruit, vegetable, and fruit juice consumption during pregnancy and childhood overweight/obesity at 4 years of age; Table S3: Associations between maternal fruit, vegetable, and fruit juice consumption during pregnancy and childhood overweight/obesity at 4 years per 100-g/day increment in intake.

## Abbreviations

The following abbreviations are used in this manuscript:

BMI	Body Mass Index
DASH	Dietary Approaches to Stop Hypertension
ECLIPSES	Ensayo CLInico Para Suplementar con hierro a EmbarazadaS
FFQ	Food Frequency Questionnaire
MedDiet	Mediterranean Diet
rMedDiet	Relative Mediterranean Diet
SGA	Small for Gestational Age
TEI	Total Energy Intake
WHO	World Health Organization
