# Supplementary material for: Maternal Consumption of Fruits, Vegetables and Fruit Juices in Relation to Offspring Weight at 4 Years
Source: Nutrients. 2026 Sep 6;18(17):2918. doi: 10.3390/nu18172918 (PMC13567673; doi:10.3390/nu18172918)
Supplement: Supplementary file 1 [file nutrients-18-02918-s001.zip › nutrients-4480096-supplementary.pdf]

Supplementary Table S1. Baseline maternal characteristics of participants included and not included in the analytical sample.

Maternal characteristics	Included (n = 252)	Not included (n = 539)	SMD[1]	p-value[2]
Age, years, mean ± SD	31.58 ± 4.55	29.76 ± 5.29	0.37	**<0.001**
Initial BMI, kg/m², mean ± SD	25.02 ± 4.53	25.05 ± 4.50	0.01	0.939
Education, n (%)			0.51	**<0.001**
Primary/Secondary	140 (55.6)	424 (78.7)		
University	112 (44.4)	115 (21.3)		
Smoking status, n (%)			0.00	0.987
Smoker	45 (17.9)	96 (17.8)		
Non-smoker	207 (82.1)	443 (82.2)		
TEI, kcal/d, mean ± SD	1756.08 ± 298.92	1785.57 ± 349.68	0.09	0.226

Values are presented as means ± SD or number (%) for continuous and categorical variables, respectively.
SMD, standardized mean difference; SD, standard deviation; BMI, body mass index; TEI, total energy intake.
[1]SMDs were used to quantify differences in baseline characteristics between participants included and not included in the analytical sample.
[2]*p*-values for differences between included and not included participants were calculated using ANOVA for continuous variables and chi-square tests for categorical variables.
Bold p-values indicate statistical significance ($p<0.05$).

Supplementary Table S2. Sensitivity analysis using inverse-probability-of-attrition weighting (IPAW) for the associations between maternal fruit, vegetable, and fruit juice consumption during pregnancy and childhood overweight/obesity at 4 years of age.

**Maternal dietary exposure**	**Tertile**	**Primary multivariable model OR (95% CI)[1]**	**IPAW-weighted model OR (95% CI)[2]**
Fruit and vegetable consumption	T1	1 (Ref.)	1 (Ref.)
	T2	0.73 (0.33–1.63)	0.92 (0.40–2.14)
	T3	**0.31 (0.13–0.74)**	**0.36 (0.14–0.93)**
Fruit consumption	T1	1 (Ref.)	1 (Ref.)
	T2	0.60 (0.27–1.33)	0.62 (0.27–1.46)
	T3	0.47 (0.21–1.03)	0.64 (0.28–1.47)
Vegetable consumption	T1	1 (Ref.)	1 (Ref.)
	T2	0.96 (0.45–2.06)	1.05 (0.46–2.41)
	T3	0.48 (0.21–1.11)	0.58 (0.24–1.40)
Total fruit juice consumption	T1	1 (Ref.)	1 (Ref.)
	T2	2.10 (0.94–4.66)	2.44 (1.02–5.84)
	T3	0.99 (0.43–2.30)	1.01 (0.41–2.52)
Natural fruit juice consumption	T1	1 (Ref.)	1 (Ref.)
	T2	0.74 (0.33–1.65)	0.75 (0.33–1.70)
	T3	0.65 (0.29–1.45)	0.64 (0.26–1.57)
Commercial fruit juice consumption	T1	1 (Ref.)	1 (Ref.)
	T2	1.34 (0.55–3.27)	1.53 (0.60–3.92)
	T3	**2.48 (1.06–5.78)**	**3.37 (1.27–8.93)**

OR, odds ratio; T, tertile; CI, confidence interval; IPAW, inverse-probability-of-attrition weighting; Ref., reference category.

[1]Primary models were adjusted for maternal age (<25, 25 to <30 or ≥30 years), maternal initial BMI (kg/m^2), maternal education level (primary/secondary or university), smoking status (yes/no), total energy intake (kcal/d), and modified 14-point rMedDiet score excluding fruits and vegetables (continuous variable).

[2]For the sensitivity analysis, stabilized inverse-probability-of-attrition weights were estimated from the probability of inclusion in the analytical sample based on baseline maternal age, initial BMI, educational level, smoking status, and total energy intake. IPAW-weighted models included the same adjustment set as the primary models and used robust variance estimation. Stabilized weights had a mean of 0.996 and ranged from 0.44 to 2.25.

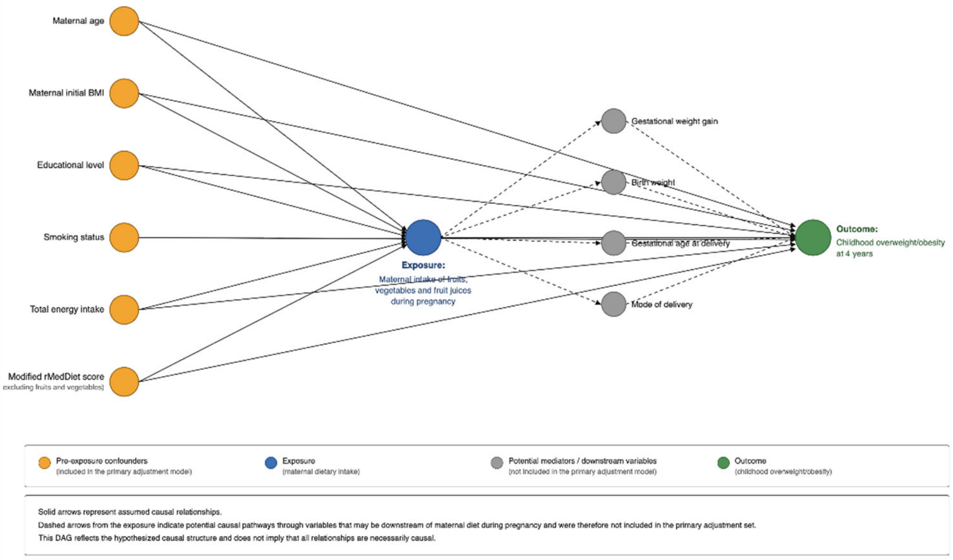

Supplementary Figure S1. Directed acyclic graph (DAG) for the association between maternal intake of fruits, vegetables, and fruit juices during pregnancy and childhood overweight/obesity at 4 years.

Supplementary Table S3. Associations between maternal fruit, vegetable, and fruit juice consumption during pregnancy and childhood overweight/obesity at 4 years per 100-g/day increment in intake.

Maternal dietary exposure	Crude OR per 100 g/day (95% CI)	p-value	Adjusted OR per 100 g/day (95% CI)	*p*-value	*p* for quadratic term[1]
Fruit and vegetable consumption	0.66 (0.43-1.02)	0.060	0.69 (0.42-1.12)	0.136	0.247
Fruit consumption	0.64 (0.37-1.10)	0.102	0.67 (0.37-1.24)	0.207	0.800
Vegetable consumption	0.53 (0.21-1.31)	0.169	0.57 (0.21-1.56)	0.277	0.196
Total fruit juice consumption	0.78 (0.49-1.23)	0.281	0.92 (0.56-1.53)	0.753	0.159
Natural fruit juice consumption	0.51 (0.27-0.96)	**0.039**	0.52 (0.25-1.08)	0.077	0.514
Commercial fruit juice consumption	1.27 (0.69-2.34)	0.445	1.67 (0.84-3.32)	0.143	0.672

OR, odds ratio; CI, confidence interval.

[1]*p*-values for the quadratic terms were obtained from the adjusted models including both the linear and quadratic terms for each dietary exposure and were used to assess potential departure from linearity.

Multivariable models adjusted for mother's age (<25, 25 to <30 or ≥30 years), mother's initial BMI (kg/m^2), mother's education level (primary/secondary or university), smoking status (yes/no), total energy intake (kcal/d) and modified 14-point rMedDiet score excluding fruits and vegetables (continuous variable).

Bold p-values indicate statistical significance ($p<0.05$).
